# Occult follicular lymphoma in a swollen regional lymph node of gastric schwannoma

**DOI:** 10.1186/s40792-020-00996-6

**Published:** 2020-09-29

**Authors:** Shuichi Fukuda, Taichi Koyama, Tomoko Wakasa, Hitoshi Hanamoto, Tomoyuki Tsujimoto, Atsushi Gakuhara, Hideo Tomihara, Katsuya Ohta, Kotaro Kitani, Kazuhiko Hashimoto, Hajime Ishikawa, Jin-ichi Hida, Masao Yukawa, Yoshio Ohta, Masatoshi Inoue

**Affiliations:** 1 Department of Gastroenterological Surgery, Kindai University Nara Hospital, 1248-1, Otoda-cho, Ikoma, Nara 630-0293 Japan; 2 Department of Pathology, Kindai University Nara Hospital, Nara, Japan; 3 Department of Hematology, Kindai University Nara Hospital, Nara, Japan

**Keywords:** Intraoperative pathological diagnosis, Lymphadenopathy, Lymphoid cuff, Malignant lymphoma, Submucosal tumor

## Abstract

**Background:**

Regional lymphadenopathy is more commonly noted in gastric schwannomas than in other gastric submucosal tumors. Most of the swollen lymph nodes associated with gastric schwannomas are non-metastatic lymphadenopathy.

**Case presentation:**

A 69-year-old Japanese woman was referred to our hospital with a chief complaint of abdominal discomfort. Contrast-enhanced computed tomography (CT) of the abdomen revealed an extraluminal tumor with heterogeneous enhancement at the middle stomach on the lesser curve, accompanied with one swollen lymph node approximately 10 mm in size and several small lymph nodes in the perigastric region. These lymph nodes were flat; therefore, we considered them to be non-metastatic. The main tumor was removed via wedge resection. Soft and slightly swollen lymph nodes, which were compatible with the lymph nodes noted in the preoperative CT, were found near the main tumor in the fatty tissue at the lesser curvature of the stomach. An excisional biopsy of the largest lymph node was performed for the diagnosis. Based on pathological findings, a diagnosis of gastric schwannoma and follicular lymphoma (FL) was confirmed. The patient is doing well without recurrence of either the gastric schwannoma or FL 28 months postsurgery.

**Conclusions:**

The present report detailed an extremely rare case of FL coincidentally discovered in the swollen regional lymph node of gastric schwannoma.

## Background

Gastric schwannomas are rare mesenchymal tumors, accounting for approximately 0.2% of all gastric tumors [1]. Recent studies reported that a computed tomography (CT) finding of gastric schwannoma demonstrated regional lymphadenopathy more frequently than other gastric submucosal tumors (SMTs): gastric schwannoma, 29.0% (≥ 20 mm) and 75.0% (≥ 50 mm); gastric gastrointestinal stromal tumor, 3.5% (≥ 20 mm) and 5.1% (≥ 50 mm); and gastric leiomyoma, 0.0% (both ≥ 20 mm and ≥ 50 mm) [2, 3]. The majority of schwannomas are benign; only a very small proportion is considered malignant with a potential to metastasize [4]. Among the gastric schwannoma patients with swollen lymph nodes, it is extremely rare to actually present with pathological lymph node metastasis [5–7]. Thus, the majority of swollen lymph nodes associated with gastric schwannomas are non-metastatic lymphadenopathy. Here we present an extremely rare case of a patient with a follicular lymphoma (FL) coincidentally discovered in the swollen regional lymph node of gastric schwannoma.

## Case presentation

A 69-year-old Japanese woman was referred to our hospital with a chief complaint of abdominal discomfort. She had no history of smoking and was a social drinker. She also had a history of hyperlipidemia, depression, and uterine myoma. Body temperature was normal. Physical examination revealed no swelling of superficial lymph nodes. The patient had regular bowel movements with normal stools. She had no B symptoms, including fever, night sweats, or weight loss. Laboratory data were unremarkable: white blood cell (WBC) count, 5890/µL; hemoglobin, 13.2 g/dL; platelet count, 254,000/µL; and lactate dehydrogenase (LDH) level, 217 U/L. Tumor markers, including carcinoembryonic antigen and carbohydrate antigen 19-9, were within normal ranges. The serum soluble interleukin-2 receptor (sIL-2R) level was also within normal ranges.

**Fig. 1:**
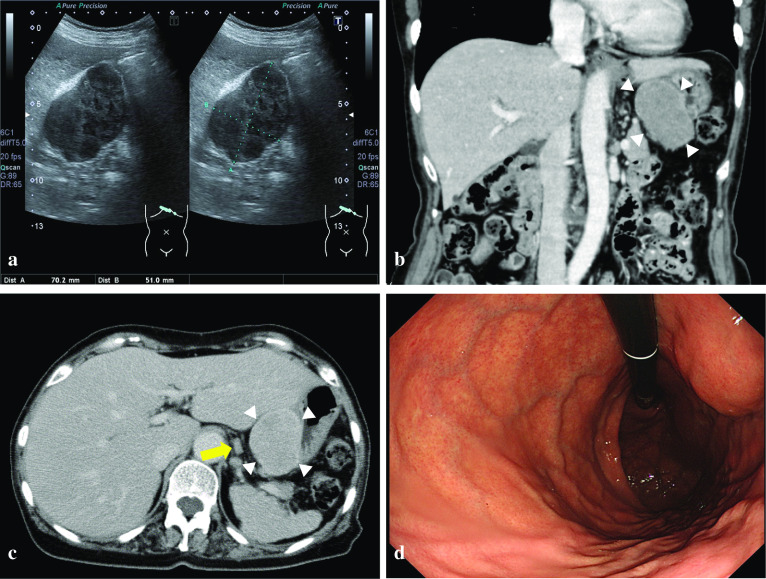
**a** Abdominal ultrasonography revealing a well-circumscribed hypoechoic mass, contiguous with the proper muscle layer, approximately 70 mm in size, located at the middle stomach on the lesser curve. **b**, **c** Contrast-enhanced CT of the abdomen showing an extraluminal tumor with heterogeneous enhancement at the middle stomach on the lesser curve (white arrowheads), accompanied with one swollen lymph node approximately 10 mm in size (yellow arrow) and several small lymph nodes in the perigastric region. **d** Endoscopy showing compression of the anterior wall of the gastric body along with normal mucosa

Abdominal ultrasonography revealed a well-circumscribed hypoechoic mass, contiguous with the proper muscle layer, approximately 70 mm in size, located at the middle stomach on the lesser curve (Fig. 1a). Contrast-enhanced CT of the abdomen demonstrated an extraluminal tumor with heterogeneous enhancement at the middle stomach on the lesser curve, accompanied with one swollen lymph node approximately 10 mm in size and several small lymph nodes in the perigastric region (Fig. 1b, c). These lymph nodes were flat; therefore, we considered them to be non-metastatic. No liver metastasis, peritoneal dissemination, pleural fluid, ascites, or splenomegaly was observed. Endoscopy indicated compression of the anterior wall of the gastric body along with normal mucosa (Fig. 1d). Several biopsy specimens were taken, which showed evidence of erosive hyperplastic gastritis. Endoscopic ultrasonography and endoscopic ultrasound-guided fine needle aspiration were not performed for the diagnosis because a SMT greater than 50 mm in size is an indication for surgery regardless of the preoperative definitive diagnosis [8].

Surgical exploration of the abdomen revealed no evidence of ascites or metastasis to the liver or peritoneum. An extraluminal tumor arising from the middle body of the stomach on the lesser curve was noted. The tumor was removed via wedge resection. Soft and slightly swollen lymph nodes, which were compatible with the lymph nodes noted in the preoperative CT, were found near the main tumor in the fatty tissue at the lesser curvature of the stomach. An excisional biopsy of the largest lymph node, likely compatible with the swollen lymph node seen in the preoperative CT, was performed for the diagnosis. At this point, we believed these lymph nodes to represent reactive lymphadenopathy.

**Fig. 2:**
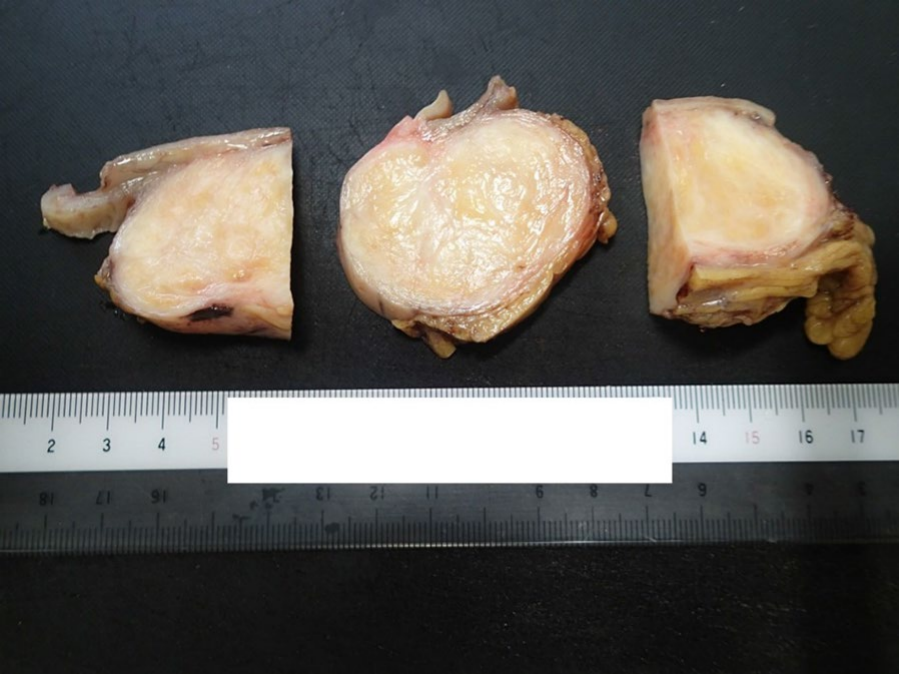
The cut surface of the tumor revealing a well-circumscribed yellowish-white solid mass

**Fig. 3:**
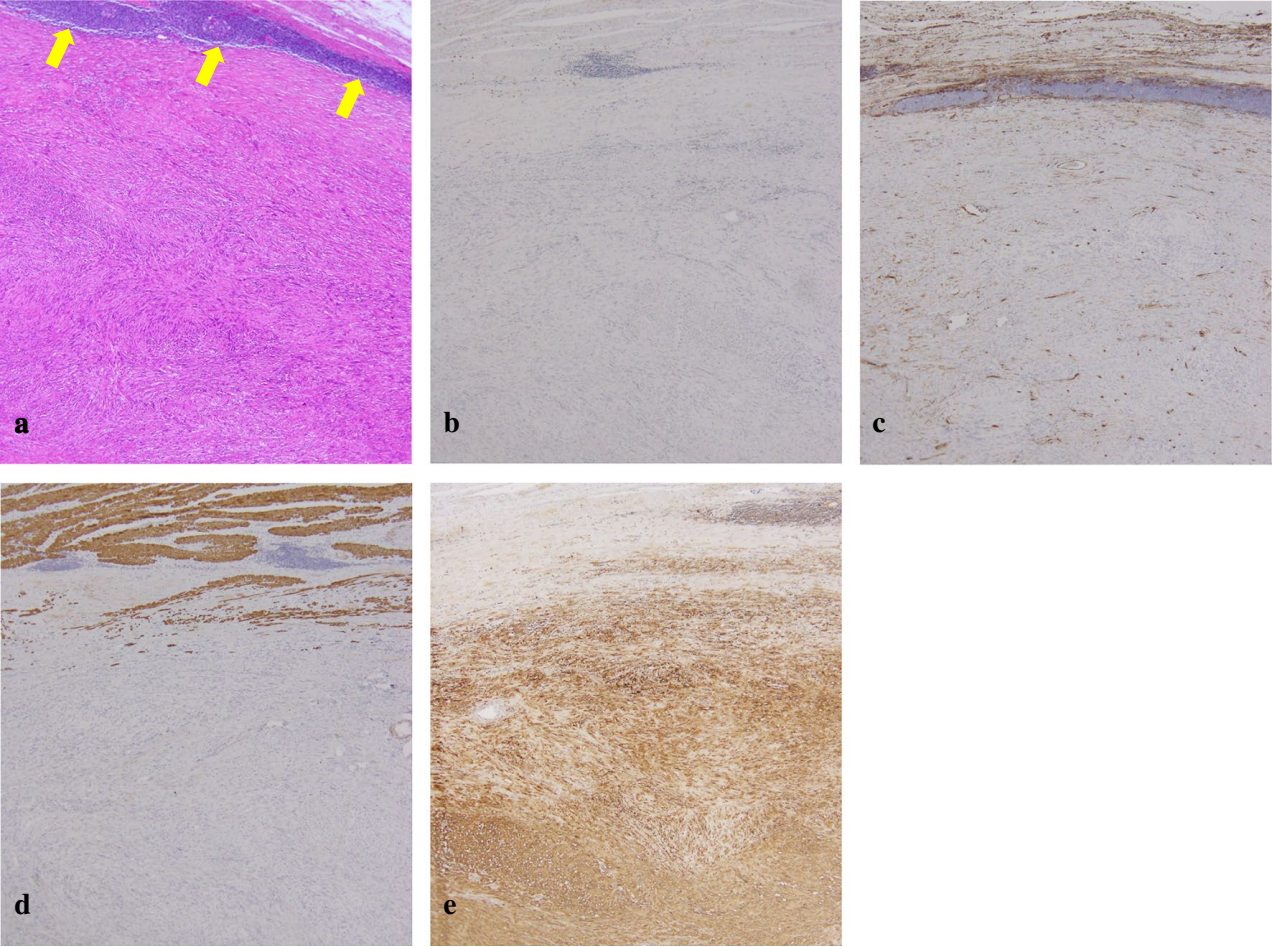
**a** Hematoxylin–eosin staining demonstrating a bundle-like growth of the spindle-shaped tumor cells with acidophilic cytoplasm. A peritumoral lymphoid cuff is recognized (yellow arrows). **b**–**e** Immunohistochemical staining revealing that the tumor is negative for KIT (**b**), CD34 (**c**), and desmin (**d**) and positive for S-100 protein (**e**)

**Fig. 4:**
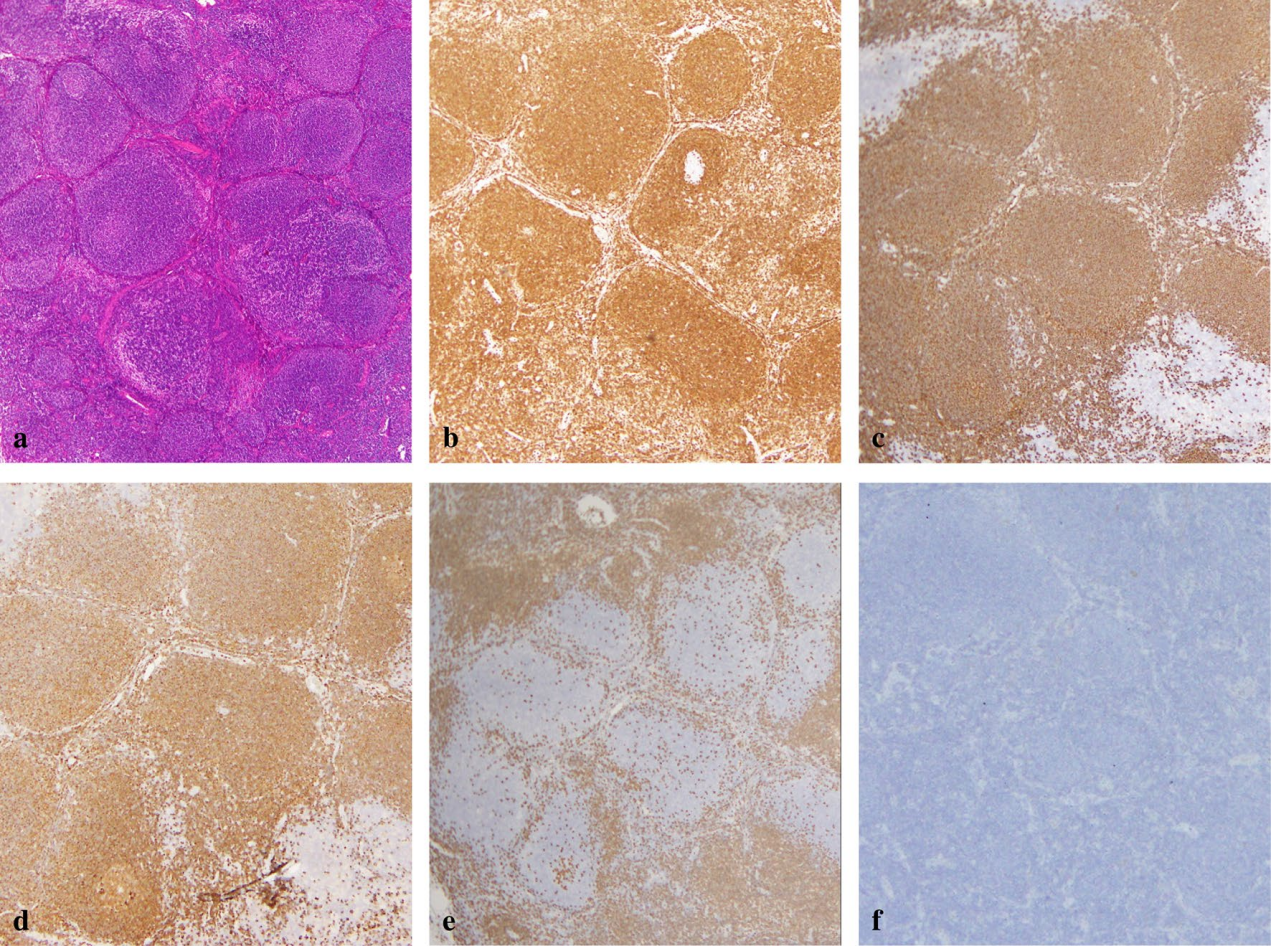
**a** Hematoxylin–eosin staining of the lymph node showing nodular proliferation of atypical lymphoid cells composed predominantly of centrocytes with admixed scattered centroblasts. **b**–**f** Immunohistochemical staining revealing that the lymph node is positive for bcl-2 (**b**), CD20 (**c**), and CD79a (**d**) and negative for CD3 (**e**) and CD30 (**f**)

The resected specimen was 80 × 60 × 50 mm in size. The tumor grew exophytically. The cut surface of the tumor revealed a well-circumscribed yellowish-white solid mass (Fig. 2). Hematoxylin–eosin staining disclosed a bundle-like growth of the spindle-shaped tumor cells with acidophilic cytoplasm (Fig. 3a). These were composed predominantly of tumor cells arranged haphazardly (Antoni type B) and secondarily of tumor cells with nuclear palisading (Antoni type A). A peritumoral lymphoid cuff was recognized (Fig. 3a). The mitotic count was 0 to 1 per 50 high-power fields (HPFs). The tumor contained no necrosis and atypical mitosis was not identified. The resection margins were free of tumor cells. Immunohistochemical staining revealed that the tumor was negative for KIT, CD34, and desmin and positive for S-100 protein (Fig. 3b–e). The MIB-1 labeling index of the tumor cells was 1% to 2%. Hematoxylin–eosin staining of the lymph node showed nodular proliferation of atypical lymphoid cells composed predominantly of centrocytes with admixed scattered centroblasts (Fig. 4a). Immunohistochemical staining revealed that the lymph node was positive for bcl-2, CD20, and CD79a and negative for CD3 and CD30 (Fig. 4b–f). These histopathological and immunohistochemical findings were consistent with a gastric schwannoma and FL. The centroblasts were noted 2 to 3 per 50 HPFs; therefore, histological grading of FL was Grade 1 [9].

The patient had an uneventful postoperative course and was discharged from our hospital on postoperative day 9. Postoperative positron emission tomography (PET) revealed no significant accumulation of ^18^F-fluorodeoxyglucose. Bone marrow aspiration indicated no bone marrow invasion of FL. The patient was diagnosed with a grading of stage I according to the Ann Arbor classification. The Groupe d’Etude des Lymphomes Folliculaires (GELF) criteria were applicable as low-tumor burden FL and the patient was asymptomatic; therefore, the watchful waiting approach was decided upon as follow-up [9]. The patient is doing well without recurrence of either the gastric schwannoma or FL 28 months postsurgery.

## Discussion

Schwannomas are neurogenic tumors originating from Schwann cells of a peripheral nerve sheath. They rarely occur in the gastrointestinal tract, with the stomach being the most common location. Gastric schwannomas are normally benign, and malignant transformation, with potential for lymph node metastasis, is extremely rare [4]. The majority of the swollen lymph nodes associated with gastric schwannomas are non-metastatic lymphadenopathy. In this paper, we report the case of a patient with a swollen lymph node of gastric schwannoma diagnosed with FL.

In the present study, the resected tumor histologically showed a peritumoral lymphoid cuff, which is known as a specific morphological finding of schwannoma, present in 78.8%–96.0% of gastric schwannomas [10–12]. Hou et al. speculate that the lymphoid cuff may be the result of cytokines secreted by the tumor cells that induce chemokinesis of lymphocytes [13]. This theory is supported by a recent paper; Bae et al. reported that a peritumoral lymphoid cuff of the main tumor positively correlates with the presence and size of regional lymphadenopathy in gastric schwannomas [12]. Regional lymphadenopathy is more commonly noted in gastric schwannomas than in other gastric SMTs [2, 3].

Malignant lymphoma, including FL, is usually noticed by the appearance of B symptoms and/or cervical, axillary, or inguinal swollen lymph nodes [14]. After the imaging test, malignant lymphoma is diagnosed by the excisional biopsy of a cervical, axillary, mesenteric, inguinal, mediastinal, or para-aortic swollen lymph node. Elevation of serum LDH and sIL-2R level, and abnormality of WBC count are helpful for diagnosis. Bulky lymphadenopathy growing around vessels, called the “sandwich sign,” is known as a specific CT feature of the advanced malignant lymphoma [15]. In the present study, FL was coincidentally diagnosed, although there were no B symptoms, blood test abnormality, or significantly swollen lymph nodes on CT.

Primary lesion resection is usually performed for the gastric schwannoma; however, there is no consensus for the operative procedure of gastric schwannoma with swollen lymph nodes. In clinical practice, gastrectomy with lymph node dissection is occasionally performed when swollen lymph nodes are accompanied with gastric schwannomas, due to concern about the possibility of metastasis from the main tumor. However, most cases actually have no pathological lymph node metastasis [5–7]. This means that the routine lymph node dissection for the swollen lymph nodes of gastric schwannoma is unnecessary in most cases and can be an excessive invasion. Lymph node dissection is not necessary when the swollen lymph node is either non-metastatic or malignant lymphoma. From a clinical perspective, to avoid excessive invasion, the lymph node’s metastatic potential should be determined prior to nodal dissection. If instant diagnosis of malignant lymphoma can become enabled in the future, we propose that the patients undergo intraoperative pathological diagnosis for swollen lymph nodes of gastric SMTs, including schwannoma. This may make it possible to avoid unnecessary lymph node dissection.

In the present case, the patient was asymptomatic and coincidently presented with Ann Arbor stage I and low-tumor burden FL. The patient received no initial therapy for FL. FL is the most common indolent B-cell non-Hodgkin’s lymphoma, most of which are diagnosed in an advanced stage (Ann Arbor stage III/IV) [14, 16]. Limited stage (Ann Arbor I/II) FL is relatively uncommon; therefore, high-quality evidence for the treatment of limited stage FL is lacking [16]. Previous reports in the United States showed that the initial therapy given for stage I patients was as follows: rituximab plus chemotherapy, 28%; radiation therapy, 27%; observation, 17%; systemic therapy plus radiation therapy, 13%; rituximab monotherapy, 12%; and other, 3% [17]. Selected limited stage patients, such as asymptomatic and low-tumor burden patients, can be candidates for the strategy of a watchful waiting approach [9, 14]. This is consistent with our present case. Advani et al. reported that stage I/II patients who received no initial therapy demonstrated that 63% of patients did not require treatment at a median follow-up of 86 months, and the estimated survival at 10 years was 85% [18]. Friedberg et al. reported that stage I patients managed with no initial therapy did not have a worse progression-free survival than those managed with radiation therapy [17].

The prognosis for an individual FL patient can be estimated by the Follicular Lymphoma International Prognostic Index (FLIPI), which is based on clinical and laboratory findings [9]. Five adverse prognostic factors of FLIPI were selected: age (> 60 years vs ≤ 60 years), Ann Arbor stage (III/IV vs I/II), hemoglobin level (< 12 g/dL vs ≥ 12 g/dL), number of nodal areas (> 4 vs ≤ 4), and serum LDH level (above normal vs normal or below). Three risk groups were defined: low (0–1 adverse factor), intermediate (2 adverse factors), and poor (≥ 3 adverse factors). In our case, one adverse factor (> 60 years) was applicable and the risk group was low. On the other hand, gastric schwannomas are normally benign; even those over 100 mm in size and with a mitotic rate greater than 5/50 HPFs showed no evidence of aggressive behavior [10]. The benign gastric schwannoma rarely recurs after complete surgical resection [19]. In this case, the patient was diagnosed with a gastric schwannoma 80 mm in size with the mitotic count 0 to 1 per 50 HPFs, which was completely resected. Concerning the FL and gastric schwannoma in this case, long-term survival is expected to be good.

As this was a single-patient case report, these findings need to be confirmed by the accumulation of prospective evidence from more patients in multiple institutions. Gastric schwannomas are relatively rare; therefore, the number of patients treated in a single institution is limited. However, the current findings provide important information that can contribute to the development of a treatment strategy for gastric schwannomas accompanied with swollen lymph nodes.

## Conclusions

The present report detailed an extremely rare case of FL coincidentally discovered in the swollen regional lymph node of gastric schwannoma. Gastric schwannomas are often accompanied by swollen regional lymph nodes, and many surgeons are confused regarding the need for lymph node dissection for such cases. This case indicates that when encountering a gastric schwannoma with regional lymphadenopathy, surgeons need to consider the possibility of malignant lymphoma and refrain from performing unnecessary lymph node dissection.

## Data Availability

All data generated or analyzed during this study are included in this published article.

## Abbreviations

CT: Computed tomography
FL: Follicular lymphoma
FLIPI: Follicular lymphoma international prognostic index
HPF: High-power field
LDH: Lactate dehydrogenase
PET: Positron emission tomography
sIL-2R: Soluble interleukin-2 receptor
SMT: Submucosal tumor
WBC: White blood cell
